# Combined strategy of α 9-integrin transduction and AEIDGIEL peptide-functionalized fibrin gel biomaterials to promote mature DRG neurite growth

**DOI:** 10.3389/fncel.2025.1568004

**Published:** 2025-04-01

**Authors:** Anda Cimpean, Lars Roll, Jacqueline Reinhard, Jessica C. F. Kwok, Andreas Faissner, Fred de Winter, James W. Fawcett, Pavla Jendelová

**Affiliations:** ^1^Institute of Experimental Medicine, Czech Academy of Science, Prague, Czechia; ^2^Second Faculty of Medicine, Charles University, Prague, Czechia; ^3^Department of Cell Morphology and Molecular Neurobiology, Faculty of Biology and Biotechnology, Ruhr University Bochum, Bochum, Germany; ^4^Faculty of Biological Sciences, University of Leeds, Leeds, United Kingdom; ^5^Neuroregeneration Research Group, Netherlands Institute for Neuroscience, Amsterdam, Netherlands; ^6^Department of Clinical Neurosciences, John Van Geest Centre for Brain Repair, University of Cambridge, Cambridge, United Kingdom

**Keywords:** α9-integrin, fibrin gel, AEIDGIEL peptide, tenascin-c, dorsal root ganglion, neurite growth, spinal cord injury, biomaterials

## Abstract

**Introduction:**

Spinal cord injury involves complex pathobiological mechanisms, necessitating a multidimensional approach for its cure. Previous studies have shown that α9-integrin expression and activation in mature dorsal root ganglion neurons enable the regeneration of injured axons within the spinal cord. However, tissue cavitation and fibrosis impede the regenerating axons from following their usual pathways, forcing them to seek alternative routes rich in tenascin-C, the primary ligand of the integrin. Fibrin gel, an FDA-approved and biocompatible material, can offer three-dimensional support for axonal extension through the cavitated area, thus preventing the formation of aberrant paths and connections that occur in the absence of a suitable scaffold.

**Methods:**

The aim of this study was to investigate how combining α9-integrin expression by adeno-associated virus with the use of a fibrin gel as an extracellular microenvironment affects the growth of mature DRG neurites *in vitro*. Additionally, we sought to functionalize fibrin with integrin ligand peptides, specifically AEIDGIEL, the active domain of tenascin-C, to ensure α9-integrin activation.

**Results:**

Our results indicate that fibrin gels are a suitable biomaterial for promoting neurite growth and that AEIDGIEL peptide effectively activates the integrin. Furthermore, we corroborate an autocrine signaling loop of α9-integrin and TN-C produced by neurons.

**Discussion:**

the proposed combination therapy of α9-integrin and fibrin gel biomaterials incorporating AEIDGIEL peptide shows promise for addressing the complex challenges of spinal cord injury and promoting effective neural regeneration, laying the foundation for further *in vivo* research.

## 1 Introduction

Spinal cord injury (SCI) can result from either traumatic events such as vertebral fractures or non-traumatic causes like infections or vascular damage, and has a poor prognosis with sensory, motor and autonomic disfunction, which leads to disability and imposes a heavy burden on healthcare systems and society (Ding et al., 2022). SCI creates a complex pathological situation with many barriers to recovery, including the inability of the adult central nervous system (CNS) axons to regenerate, cavitation and excessive scarring at the lesion site. Therefore, treatment requires a combinatorial approach with different therapies acting in a synergistic manner. Currently, achieving complete recovery from severe SCI remains challenging due to the absence of successful translational therapies (Fawcett, 2020; Hu et al., 2023). Dorsal root ganglion (DRG) neurons are the afferent neurons that relay sensory information from the periphery to the brain. Injured central dorsal root axons are unable to regenerate in the spinal cord resulting in loss of sensorimotor functions and neuropathic pain. Previous studies reported that α9-integrin expression and activation in mature DRG neurons allow regeneration of injured axons into the spinal cord and enable functional recovery of sensation and locomotion (Andrews et al., 2009; Cheah et al., 2016; Stepankova et al., 2023). The major ligand of the integrin is tenascin-C (TN-C), the main extracellular matrix inhibitory glycoprotein upregulated in the CNS environment after SCI. The bioactive domain of TN-C that activates α9-integrin is the AEIDGIEL sequence, contained in the B-C loop of the third subunit of fibronectin type III protein (Yokosaki et al., 1998). Integrin expression provides a proper adhesion receptor to control migration of axon growth through the unfavorable environment, and triggers an intracellular genetic program specific to CNS regeneration, with upregulation of genes related to ubiquitination, autophagy, endoplasmic reticulum, casein kinases, transport/trafficking and other signaling molecules (Cheah et al., 2023). However, the massive tissue disruption and extensive scarring formation at the injury side hinder axons capable of regeneration and prevent the formation of physiological meaningful connections after injury. Hydrogel materials can expand to fill the entire wound site, providing a surface and scaffold through which nerves can regenerate, avoid aberrant growth and protect themselves from the surrounding fibrosis. Many studies have demonstrated their beneficial role in SCI, however, hydrogel treatment alone doesn’t completely restore neurological function (Cai et al., 2023). Hydrogel materials contain extracellular matrix (ECM) proteins such as fibrin, which plays an important role in natural wound healing. Because of its affordability, cytocompatibility and ability to modulate angiogenesis and inflammation, fibrin has been used clinically for wound coverings, surgical glues and cell delivery (Jarrell et al., 2021).

The aim of this study was to examine how the combination of α9-integrin expression and the use of a fibrin gel as an extracellular microenvironment affects the growth of mature DRG neurites *in vitro*. Additionally, we aimed to improve the biomaterial properties by incorporating the bioactive TN-C domain, AEIDGIEL, to ensure integrin activation. We assessed neurite growth of un-transduced, GFP-transduced and α9-integrin-transduced DRG neurons cultured on coverslips with various coatings, fibrin gels and AEIDGIEL-modified fibrin gels. We concluded that fibrin gels serve as a suitable biomaterial to promote neurite growth and that AEIDGIEL peptide is sufficient to activate the integrin. The intriguing finding that α9-integrin-transduced neurons grow long neurites irrespective of the surface motivated us to further investigations which led to the corroboration of the autocrine signaling loop of α9-integrin and TN-C produced by neurons.

## 2 Materials and methods

### 2.1 Cell culture

Adult 3 months old WT C57BL/6 and TN-C KO mice (kindly provided by Andreas Faissner, Ruhr University Bochum, Bochum, Germany) were placed in a close box and fully anesthetized with 3% isoflurane in air flow (Aerrane, Baxter). Once overdosed, mice were decapitated with guillotine (Ježek s.r.o., Czechia) and DRGs were extracted. All experiments were performed in accordance with the European Communities council directive of 22nd of September 2010 (2010/63/EU), follow the ARRIVE guidelines^1^ and were approved by the Ethics Committee of the Institute of Experimental Medicine CAS, Prague, Czechia. DRGs were dissociated in 0.2% collagenase (Sigma-Aldrich, C9407) for 2 h and 0.1% trypsin (Sigma-Aldrich, T0303) for 10 min in DMEM at 37°C, followed by trituration and 15% BSA (Sigma-Aldrich, A9418) gradient centrifugation. Cells were plated and grown on coated glass coverslips and fibrin hydrogel matrices for 5 days at a density of 1 × 10^4 cells/condition in the following culture medium: 1% ITS (Sigma-Aldrich, I2521), 1% PSF (Sigma-Aldrich, P4333), 10 ng/ml NGF (Sigma-Aldrich, N8133) and 0.5 μg/ml Mitomycin-C (Sigma-Aldrich, M4287) in DMEM (Thermofisher, 10566016).

### 2.2 Coating of coverslips and fibrin hydrogel matrices preparation

Coverslips (German Glass Cover Slips, 72290-08, 18 mm) were placed in 24-well plates and coated with either 20 μg/ml PDL (Sigma-Aldrich, P1149) or 1 μg/ml PDL + 10 μg/ml chicken TN-C (Sigma-Aldrich, CC115) or 1 μg/ml PDL + 5 μg/ml AEIDGIEL peptide (Sigma-Aldrich, 168312K).

Preparation of fibrin hydrogel matrices consisted of mixing a fibrinogen-based solution and an enzymatic solution in equal proportions, as described previously (Schense and Hubbell, 1999). The enzyme solution contained HEPES buffer, 2 mM CaCl_2_, 2 U/ml thrombin (Sigma-Aldrich, T6884) and 5 U/ml Factor XIII (Thermofisher, RP-43077). The enzyme solution was incubated for 10 min at 37°C on a glass coverslip, followed by the addition of the fibrinogen solution. The fibrinogen solution contained HEPES buffer, 7.5 mg/ml fibrinogen (Sigma-Aldrich, F3879), 10 μg/ml aprotinin (Sigma-Aldrich, A3428), without or with 1 mg/ml AEIDGIEL peptide. The mixture of enzyme and fibrinogen solution was incubated for 1 h at 37°C to form a polymerized gel.

### 2.3 Cell transduction

Dissociated DRG neurons were incubated with 1 × 10^9 tu/ml of AAV-CAG-GFP and AAV-CAG-α9-V5. The plasmids carrying GFP or human α9 integrin were amplified and sequenced before packaging into AAV serotype 5 (AAV5) using HEK293T cells as described previously (Verhaagen et al., 2018). Cells were incubated with the virus for 72 h at 37°C to induce viral transduction. On the third day, the media was replaced, and the cells were kept for additional 48 h to ensure protein expression.

### 2.4 Immunocytochemistry

Cells were maintained in culture for 5 days (5DIV), then fixed with 4% paraformaldehyde (PFA) for 15 min and blocked with 1% goat-serum in PBS and 2% Triton for 2 h. Cells were incubated with 1:1200 rabbit monoclonal antibody against beta III tubulin (abcam, ab68193), 1:1000 chicken polyclonal anti-GFP antibody (Thermo Fisher Scientific, A-10262) and 1:500 mouse monoclonal anti-V5 tag antibody (Thermo Fisher Scientific, R960-25) in PBS with 1% goat-serum and 2% Triton overnight at 4°C. The following day, cells were washed with PBS and 1:20 Triton and incubated for 2 h at 4°C with PBS with 1% goat-serum and 2% triton and the following secondary antibodies: 1:400 goat anti-rabbit 405 (Thermo Fisher Scientific, A-31556), 1:500 goat anti-chicken 488 (Thermo Fisher Scientific, A-11039) and 1:400 goat anti-mouse 594 (Thermo Fisher Scientific, A-11032). Cells were washed several times with PBS and Triton 1:20 and coverslips were mounted with Fluorosave (Calbiochem).

### 2.5 Microscopy, neurite number, outgrowth and branching assay and statistical analysis

Cells were analyzed by fluorescent microscopy. Imaging was performed using a Zeiss Axio Observer D1 fluorescence microscope equipped with inverted phase contrast system. Since the cells were cultured on single-plane surfaces, all images were captured as single-plane (no z-stack) at 40 × magnification. Consistent imaging parameters were maintained across all conditions (exposure time of 200 ms, laser power at 20%, and fixed gain settings). To assess viral transduction, cells were identified and categorized based on their fluorescence signals: green for AAV-GFP-transduced cells, red for AAV-α9-V5-transduced cells, and only blue for un-transduced cells. The longest neurite length per neuron and the number of neurites originating from the cell body were measured manually using ImageJ software (National Institutes of Health). Branching analysis was conducted automatically using Scholl analysis via the Neuroanatomy plugin in ImageJ. The plugin created concentric circles (Scholl shells) placed 10 μm apart, with the cell body in the middle and counts the points, termed intersections, where the processes intersect the Scholl shells. These intersections were visually represented by different colors in the figure based on the specific Scholl shell they intersected. Data were quantified from at least three separate experiments. Comparisons were made among un-transduced (NT), AAV-GFP-transduced (GFP), and AAV-α9-integrin-transduced (A9) DRG cultures under different growth conditions. Statistical analysis was performed using GraphPad Prism 9. Normality was assessed with the D’Agostino and Pearson test. Non-normally distributed data were analyzed using the Kruskal-Wallis test with Dunn’s multiple comparisons, while normally distributed data were evaluated with a one-way ANOVA for neurite outgrowth and a two-way ANOVA for neurite number and branching, followed by Tukey’s *post-hoc* test.

## 3 Results

### 3.1 Expression of α9-integrin in adult DRG neurons enables neurite growth on tenascin-C and its motif peptide AEIDGIEL

**FIGURE 1:**
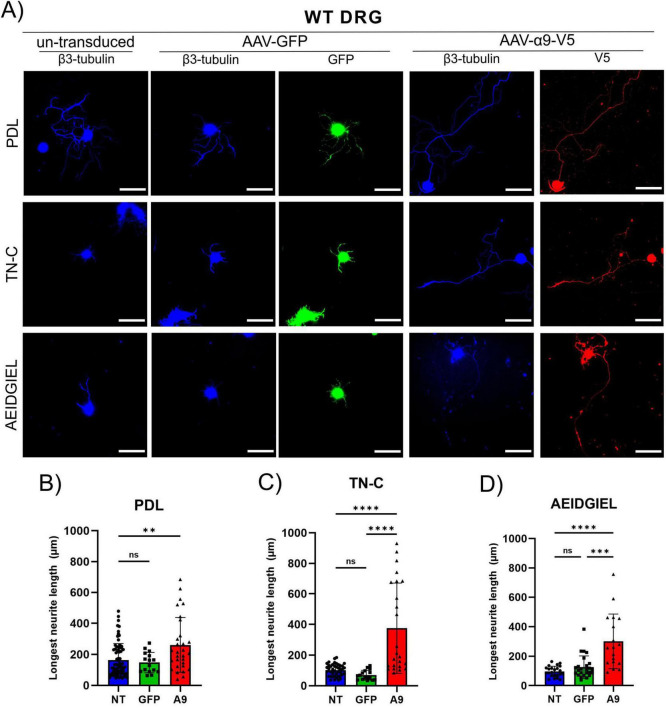
WT dorsal root ganglion (DRG) neurite growth on treated coverslips. **(A)** Un-transduced, GFP-transduced and α9-integrin-transduced neurons growing on PDL-only, TN-C or AEIDGIEL coated coverslips. Quantification of the longest neurite (μm) growing on **(B)** PDL-coated coverslips, **(C)** Tenascin-C (TN-C) coated coverslips, **(D)** AEIDGIEL coated coverslips. The longest neurite of each neuron was quantified from at least three separate experiments, with data representing individual neurites. **p < 0.01, ***p < 0.001, ****p < 0.0001. Error bars indicate SD. Scale bars, 70 μm.

Previous studies have shown that α9-integrin expression in adult DRG neurons enhances neurite outgrowth and overcomes the growth inhibition by TN-C, a major extracellular inhibitory protein that is upregulated after SCI (Andrews et al., 2009; Cheah et al., 2016). α9-integrin recognizes a peptide in the third fibronectin type III domain of TN-C, AEIDGIEL (Yokosaki et al., 1998). First, we confirmed that TN-C inhibits neurite outgrowth from adult DRG neurons, and then determined whether the presence of the AEIDGIEL peptide creates an inhibitory environment similar to wild-type TN-C. We cultured adult DRG neurons on glass coverslips coated with PDL alone, with chicken TN-C, or with the AEIDGIEL peptide. Cells were transduced with AAV-CAG-GFP and AAV-CAG-α9-V5 (Figure 1A). On PDL coated coverslips, un-transduced and GFP-transduced DRG neurons grew to an average length of 150 μm (Figure 1B), whereas on coverslips coated with TN-C or AEIDGIEL the average neurite outgrowth was 90 μm (Figures 1C, D), significantly lower (Supplementary Figure 1). Next, we asked whether AAV-mediated expression of α9-integrin would promote outgrowth on TN-C and AEIDGIEL. Cells with α9-transduced neurites grew significantly longer than controls on the surface of both TN-C and AIDGIEL, with an average length of 260 μm (Figures 1C, D). However, the length of neurites grown by α9-transduced neurons on coverslips coated with PDL alone also increased compared to the controls (Figure 1B), and the length was not significantly different to that of neurons grown on TN-C or AEIDGIEL (Supplementary Figure 2A). α9-integrin thus, promoted growth on all surfaces. Because α9-integrin is a specific receptor for TN-C it is likely that axons respond to TN-C in all three situations. The TN-C protein has been shown to be produced by several glial cell types, as well as by DRG neurons, where it is located on their membrane and the surrounding culture surface (Andrews et al., 2009). Therefore, from this set of experiments, it is not possible to determine whether α9-transduced neurons are responding to the TN-C or AEIDGIEL that has been applied to the culture surface, or the TN-C secreted by DRG neurons.

### 3.2 α9-integrin activation relies on neuronal tenascin-C

**FIGURE 2:**
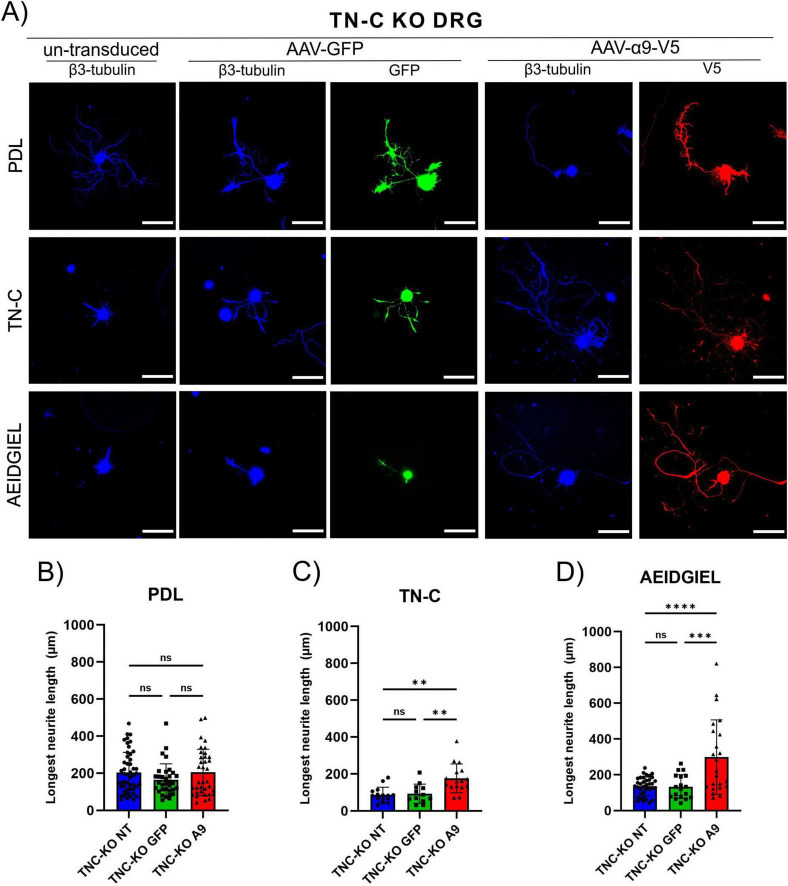
Tenascin-C (TN-C) KO dorsal root ganglion (DRG) neurite growth on treated coverslips. **(A)** Un-transduced, GFP-transduced and α9-integrin-transduced TN-C knockout neurons growing on PDL-only, TN-C or AEIDGIEL coated coverslips. Quantification of the longest neurite (μm) growing on **(B)** PDL-coated coverslips, **(C)** TN-C coated coverslips, **(D)** AEIDGIEL coated coverslips. The longest neurite of each neuron was quantified from at least three separate experiments, with data representing individual neurites. **p < 0.01, ***p < 0.001, ****p < 0.0001. Error bars indicate SD. Scale bars, 70 μm.

In the above results, we showed that α9-integrin-transduced DRG neurons grew on coverslips coated with TN-C or AEIDGIEL to an average neurite length of 260 μm, but they also grew to a similar distance on PDL surfaces without added TN-C or peptide. This suggests that cells in culture produce TN-C protein, that adheres to PDL and provides a ligand for α9-integrin. Therefore, to confirm that α9-integrin-transduced DRG neurites can grow on AEIDGIEL alone, we had to prevent TN-C production by cultured DRGs and therefore repeated the neurite outgrowth experiments with dissociated cultures of TN-C knockout DRGs (Steindler et al., 1995). The three experimental surfaces were coverslips coated with PDL, followed by one group coated with AEIDGIEL and the other with chicken TN-C. On these three surfaces, un-transduced and GFP- or α9-transduced adult DRG neurons from TN-C knockout mice were grown (Figure 2A). Un-transduced, GFP-transduced and α9-transduced TN-C knockout cells growing on PDL coated coverslips showed equal and small neurite outgrowth (Figure 2B). TN-C knockout α9-transduced neurons growing on PDL coated coverslips showed significantly less neurite growth than WT α9-transduced neurons (Supplementary Figure 2B). Integrin-transduced TN-C knockout cells grew on TN-C and AEIDGIEL coated coverslips neurites two to three times longer than un-transduced or GFP-transduced (Figures 2C, D). This demonstrates that α9-transduced TN-C knockout neurons do not show enhanced axon growth in the absence of ligand on the culture surface. However, in TN-C knockout neurons, TN-C or AEIDGIEL coated on the surface of the PDL can strongly promote the growth of α9-integrin-transduced neurites. These results demonstrate that AEIDGIEL or TN-C coated on coverslips both act as a ligand for α9-integrin, and enable strong neurite outgrowth from α9-expressing DRG neurons. Comparing these results with those presented in the previous paragraph, we can conclude that WT DRGs produce enough TN-C to promote the neurite outgrowth from α9-transduced neurons. In the absence of secreted TN-C from knockout neurons, both AEIDGIEL and TN-C coated surfaces provide a good ligand for α9-integrin.

### 3.3 Fibrin gels and modified fibrin gels with AEIDGIEL promote the neurite outgrowth of α9-transduced cells

**FIGURE 3:**
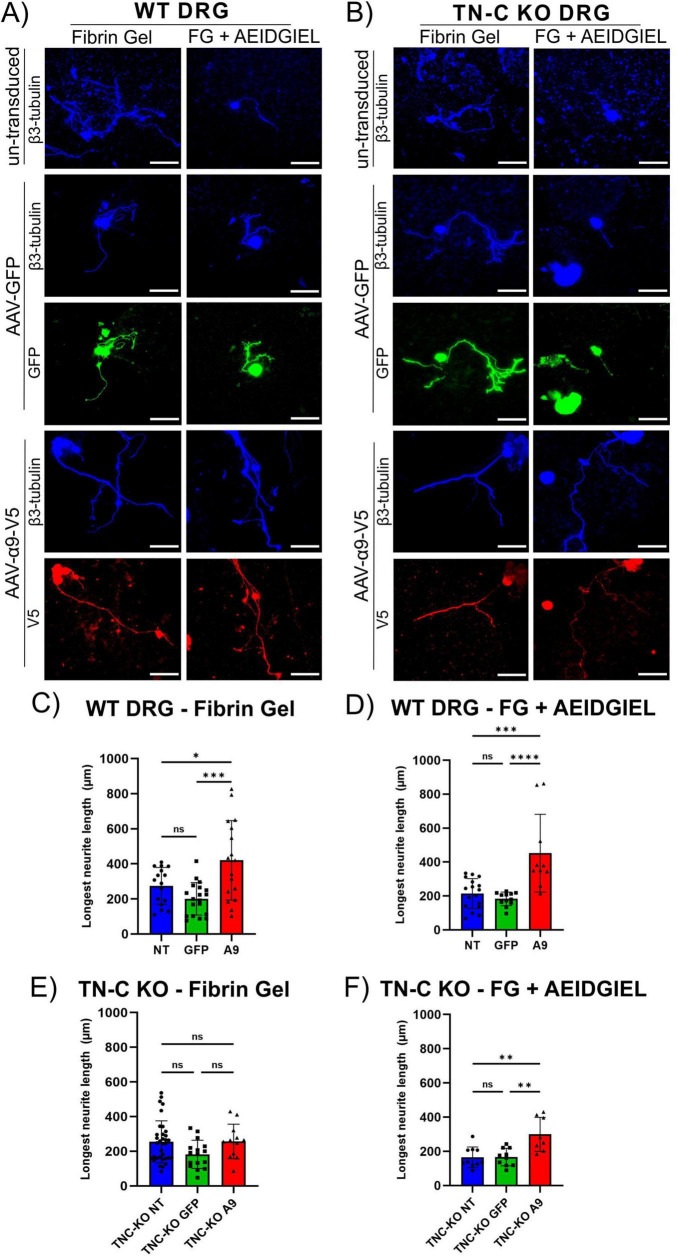
WT and tenascin–C (TN-C) KO dorsal root ganglion (DRG) neurons growing on fibrin gel and AEIDGIEL-modified fibrin gel. **(A)** Un-transduced, GFP-transduced and A9-transduced WT neurons growing on fibrin gel and fibrin gel containing AEIDGIEL peptide. **(B)** Un-transduced, GFP-transduced and A9-transduced TN-C KO neurons growing on fibrin gel and fibrin gel containing AEIDGIEL peptide. **(C)** Longest neurite length quantification (μm) of WT DRG neurons growing on fibrin gel or on **(D)** AEIDGIEL-containing fibrin gel. **(E)** Longest neurite length quantification (μm) of TN-C KO neurons growing on fibrin gel or **(F)** AEIDGIEL-containing fibrin gel. Data represents individual neurites from at least three different experiments. *p < 0.05, **p < 0.01, ***p < 0.001, ****p < 0.0001. Error bars indicate SD. Scale bars, 70 μm.

In SCI, cavitation of the injured area occurs, which physically prevents regenerating axons from passing through the lesion. Furthermore, it induces remodeling of the microenvironment of the affected area, dysregulating the ECM to a state of scarring which represents one of the major obstacles to axonal regeneration in the CNS. Biomaterials are a promising tool to overcome these limitations by providing a scaffold through which axons can grow and mimicking the natural cellular environment to prevent obstruction of neurite outgrowth. We investigated whether fibrin gels and fibrin gels containing AEIDGIEL were able to promote neurite growth driven by transduced α9-integrin (Figures 3A, B). The results show that un-transduced and GFP-transduced cells grow neurites on fibrin gel with an average length of 230 μm for both WT and TNC-KO DRG neurons (Figures 3C, E), suggesting that this biomaterial promotes physiological neurite outgrowth. As expected, α9-transduced neurons grew longer neurites in WT experiments, averaging 420 μm, due to the production of TN-C which activates the integrin (Figure 3C), but we no longer observed this effect in the TNC-KO neurons, and un-transduced, GFP-transduced and α9-transduced groups did not differ in their neurite growth (Figure 3E). TN-C knockout α9-transduced neurons growing on fibrin gel showed significantly less neurite growth than WT α9-transduced neurons (Supplementary Figure 2C).

AEIDGIEL containing fibrin gels created a slightly inhibitory environment for un-transduced and GFP-transduced cells in both WT and TNC-KO conditions, with an average neurite length of 180 μm (not significant, *p* > 0.05, Supplementary Figure 3 and Figures 3D, F). However, when DRG neurons expressed α9-integrin they grew significantly longer neurites, reaching lengths of 450 μm in WT conditions (Figure 3D) and 300 μm in TN-C KO conditions (Figure 3F).

The outgrowth of WT axons is likely mediated by one of the several integrins spontaneously expressed by DRG neurons (Werner et al., 2000; Gardiner, 2011). This growth was enhanced by the expression of α9-integrin, likely because of interaction with TN-C secreted by DRG, as discussed above. TN-C KO neurons growing on fibrin gels behaved similarly to WT except that lack of neuronal TN-C abolished the ability of α9-integrin to increase neurite length. If used as a biomaterial, the growth of α9-integrin transduced axons would be enhanced by AEIDGIEL-modified gels. This would be significant, because α9-transduced axons have the ability to re-enter CNS tissue and regenerate over long distances (Cheah et al., 2016; Stepankova et al., 2023).

**FIGURE 4:**
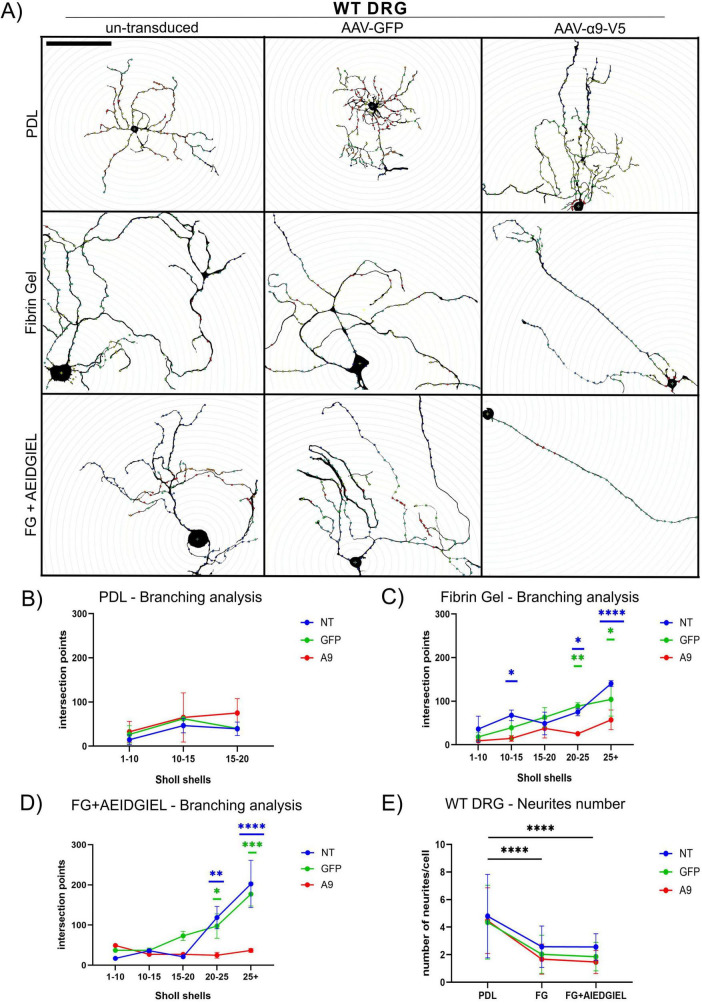
Scholl analysis of WT dorsal root ganglion (DRG) neurons growing on PDL-coated coverslips, fibrin gel and AEIDGIEL-modified fibrin gel. **(A)** Un-transduced, GFP-transduced and A9-transduced WT neurons growing on PDL-coated coverslips, fibrin gel and fibrin gel containing AEIDGIEL peptide. Graphs of the Number of intersection points depending on the interval of Scholl shells or distance from the cell body of un-transduced, GFP-transduced or a9-transduced neurons growing on **(B)** PDL-coated coverslips, **(C)** fibrin gels and **(D)** AEIDGIEL-modified fibrin gels. **(E)** Total number of neurites emerging from the cell body of un-transduced, GFP-transduced and A9-transduced WT neurons growing on PDL-coated coverslips, fibrin gels and AEIDGIEL-modified fibrin gels. Data are Mean ± SD collected from at least three different experiments. *p < 0.05, **p < 0.01, ***p < 0.001, ****p < 0.0001. Scale bars, 100 μm.

**FIGURE 5:**
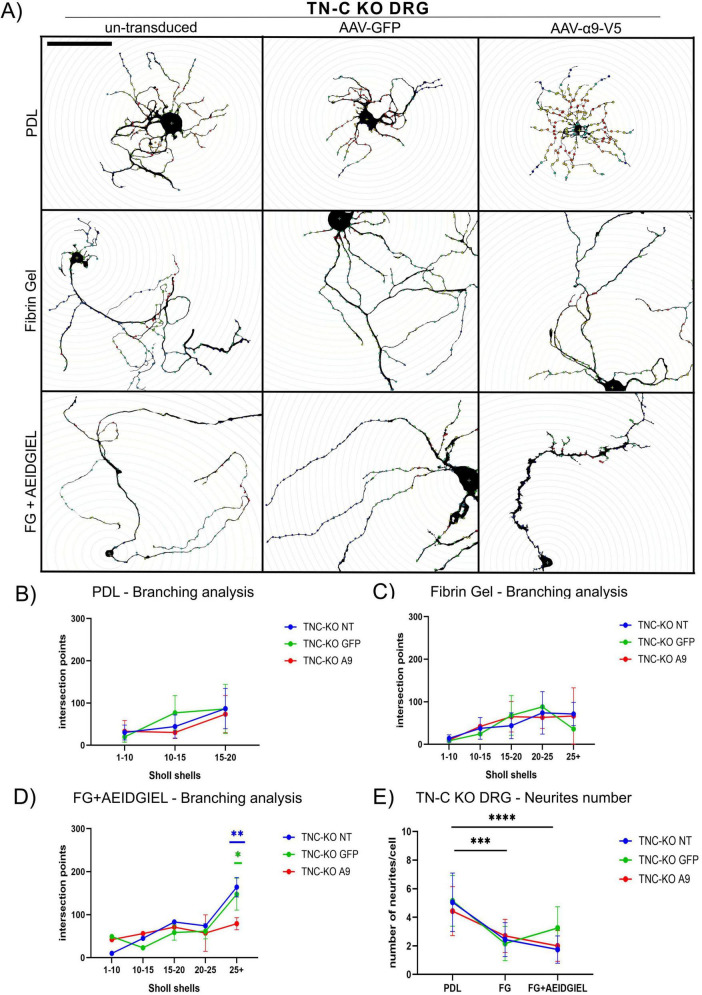
Scholl analysis of tenascin–C (TN-C) KO dorsal root ganglion (DRG) neurons growing on PDL-coated coverslips, fibrin gel and AEIDGIEL-modified fibrin gel. **(A)** Un-transduced, GFP-transduced and A9-transduced TN-C KO neurons growing on PDL-coated coverslips, fibrin gel and fibrin gel containing AEIDGIEL peptide. Graphs of the Number of intersection points depending on the interval of Scholl shells or distance from the cell body of TN-C KO un-transduced, GFP-transduced or a9-transduced neurons growing on **(B)** PDL-coated coverslips, **(C)** fibrin gels and **(D)** AEIDGIEL-modified fibrin gels. **(E)** Total number of neurites emerging from the cell body of un-transduced, GFP-transduced and A9-transduced TN-C KO neurons growing on PDL-coated coverslips, fibrin gels and AEIDGIEL-modified fibrin gels. Data are Mean ± SD collected from at least three different experiments. *p < 0.05, **p < 0.01, ***p < 0.001, ****p < 0.0001. Scale bars, 100 μm.

The favorable neurite length results prompted us to further explore neurite morphology by analyzing the number of neurites and their branching complexity through Scholl analysis (Figures 4, 5).

For both WT and TN-C KO neurons, we observed a reduction in the number of neurites emerging from the cell body when comparing cells grown on PDL-coated coverslips with those on fibrin gels or peptide-modified fibrin gels, regardless of transduction status (Figures 4E, 5E).

On PDL-coated coverslips, there were no differences in branching complexity between un-transduced and transduced cells, with all groups showing fewer than 100 intersection points on average in both WT and TN-C KO neurons (Figures 4B, 5B). When neurons were cultured on fibrin gels or fibrin gels modified with AEIDGIEL, their neurites extended further and displayed increased branching complexity, leading to a higher number of Scholl shell intersections and intersection points. However, in WT neurons, α9-integrin activation led to reduced branching complexity compared to un-transduced or GFP-transduced cells on both fibrin gel types (Figures 4C, D). In TN-C KO neurons, no significant changes in branching complexity were observed on fibrin gels due to the lack of integrin activation (Figure 5C). However, on AEIDGIEL-modified fibrin gels, where α9-integrin activation occurs, the same reduction in branching complexity was seen as in WT neurons, with α9-transduced cells showing fewer branches compared to un-transduced or GFP-transduced cells.

In summary, fibrin gel biomaterials reduced the number of neurites but promoted their outgrowth. The combination of α9-transduction, its activation, and fibrin gel resulted in fewer, longer, and straighter neurites with reduced branching. This outcome is advantageous for SCI repair, as it facilitates axonal regrowth that is directed and free of aberrant connections, enhancing the potential for functional recovery.

## 4 Discussion

Spinal cord injury affects millions of people around the world and conventional surgical and medical treatments fail to fundamentally cure it because of the complex pathological mechanisms involved that requires a multidimensional approach (Ahuja et al., 2017). To persuade axons to regenerate along the lesion we need to provide a bridge through which they can grow and trigger an appropriate intracellular response for axonal elongation. The present study investigates how a combined strategy involving the promotion of intracellular neurite growth through α9-integrin expression and the creation of an external favorable environment using fibrin gels affect DRG neurite length *in vitro*.

Neurite growth depends on the adhesion of the growth cone to the ECM and active intracellular signaling for the growth machinery. Integrins can facilitate both processes, but the ECM in an injured spinal cord lacks ligands compatible with DRG integrins, making it unfavorable for axon growth. After SCI, the ECM upregulates the TN-C protein, whose primary receptor, α9-integrin, is developmentally downregulated. Consequently, adult neurons lack this receptor to match the TN-C ligand in the injured environment, making TN-C one of the major extracellular proteins that inhibits adult axon growth. Forced expression of α9-integrin via AAV vectors has been shown to significantly promote the regeneration of adult sensory axons in the CNS after dorsal root crush (Cheah et al., 2016) and dorsal column crush (Stepankova et al., 2023). Axons expressing α9-integrin and its activator, kindlin, can grow through the non-permissive microenvironment around the lesion, which is rich in upregulated TN-C and inhibitory chondroitin sulfate proteoglycans (CSPGs). By enabling α9-integrin expression and activation, the traditionally inhibitory post-lesion environment is transformed into one that promotes axonal growth. However, tissue cavitation and fibrosis obstruct the regenerating axons from following their usual pathways, forcing them to find alternative growth routes rich in TN-C, so they extend along the meningeal connective tissue around the lesion. Some of these axons re-enter the CNS, but their regeneration pathway differs from the typical path taken by sensory axons, and they often get lost in the meninges (Stepankova et al., 2023). Hydrogels can provide a three-dimensional spatial support for axonal extension, thereby preventing the aberrant paths and connections through meninges that axons are forced to form in the absence of a suitable scaffold. The utility of hydrogels for SCI has been extensively studied and demonstrated to be able to fill the lesion area by mimicking the natural ECM, improving the microenvironment at the lesion site and promoting reconnection of damaged tissue (Cai et al., 2023). Fibrin gel is an FDA approved, biocompatible material for filling the spinal cord lesion area that prevents cavitation and facilitates DRG axons regeneration (Yu et al., 2020; Matthews et al., 2021; Pan et al., 2021; He et al., 2022). Adult DRG neurons spontaneously express several integrins that bind to ECM molecules (α3β1, α4β1, α5β1 α6β1 and α7β1), the α5β1-integrin particularly is responsible for the fibrin attachment (Werner et al., 2000; Ju et al., 2007; Gardiner, 2011). Fibrin is formed by the enzymatic polymerization of fibrinogen monomers with thrombin to form non-covalent cross-linked chains (Jarrell et al., 2021). This feature causes fibrin gel to degrade rapidly in the body, an inconvenience that limits its usefulness. However, it has been described that the addition of chemical crosslinkers and proteolysis inhibitors can improve and provide better control over the biomaterial degradation rate. The proteolytic enzyme inhibitor aprotinin has been shown to reduce fibrinolysis by inhibiting trypsin, plasmin and kallikreins, without affecting other cell functions (Coffin and Gaudette, 2016). Moreover, the addition of Factor XIII and CaCl2 solution can covalently cross-link the polymer network providing greater stability and resistance to degradation. Factor XIII has also been shown to covalently cross-link other proteins or peptides into the gel, further extending the utility of the biomaterial to include the desired bioactive domains (Schense and Hubbell, 1999). ECM molecules are notably large and challenging to construct and incorporate into the gel but short peptide domains can also act as ligands to produce the desired functions. Moreover, functionalization of fibrin with integrin ligand peptides has been shown to facilitate regeneration of neuronal precursor axons through gels (Silva et al., 2017).

We first assessed the neurite growth of adult DRG neurons on glass coverslips coated with PDL, AEIDGIEL peptide or TN-C. The results indicate that the peptide is sufficient to create the same effect as the TN-C protein, as reflected by the absence of differences in neurite growth between the two conditions: both the TN-C protein and the AEIDGIEL peptide created the expected inhibitory environment for neurite outgrowth in control cells, and α9-integrin was able to overcome it, resulting in higher average neurite length (Figures 1C, D). When DRG neurons were cultured on fibrin gels, we observed twice the neurite length in control cells than on the neutral coverslips, demonstrating that the fibrin gel is a suitable biomaterial for promoting physiological neurite growth (Figure 3C). Fibrin gel containing AEIDGIEL created a slightly but not significant inhibitory environment, which was again overcome by integrin expression (Figure 3D). The striking results showing that α9-transduced neurons grew to greater neurite length under all conditions, including a coverslip coated only with PDL (Figure 1B) or an unmodified fibrin gel (Figure 3C) wouldn’t allow us to conclude that the AEIDGIEL peptide can activate the integrin. Previous studies examining DRG neurites growth also contributed to this unexpected result, cells transfected with α9-integrin showed longer neurite outgrowth, even when grown on uncoated plastic, suggesting that the cultured cells possess α9-ligand. Andrews et al., treated neurons with TN-C siRNA, and observed a significant reduction in neurite outgrowth on uncoated plastic, but this had no effect on neurite outgrowth on TN-C or laminin coated surfaces. Furthermore, the study demonstrated TN-C immunoreactivity on DRGs bodies and processes, and TN-C deposited on surface adjacent to neurons (Andrews et al., 2009). We also observed robust outgrowth of neurites when α9-integrin is expressed regardless of the surface, leading us to conclude that neuronal TN-C activates the integrin and prevents interaction with the AEIDGIEL peptide. We therefore decided to repeat the experiments using TN-C knockout DRGs. In the knock-out condition we no longer observe significant difference in neurite growth between controls or α9-transduced cells when grown on PDL or fibrin gel, but when cells are grown on surfaces where TN-C or the AEIDGIEL peptide are exogenously provided, α9-transduced neurons show significantly higher neurite growth (Figures 2, 3E, F). This finding demonstrates that the presence of the AEIDGIEL peptide is sufficient for integrin activation and supports previous findings of an autocrine signaling loop of α9-integrin and TN-C produced by neurons, in addition to integrin interaction with extracellular ligands.

We further characterized and compared neurite number and morphology on PDL-coated coverslips, fibrin gels and AEIDGIEL-modified fibrin gels (Figures 4, 5). Our findings revealed that fibrin gels promote fewer neurites but support longer outgrowth. Additionally, the combination of fibrin gels and integrin activation resulted in less branched and more straight axons (Figures 4C, D, 5D). This configuration is ideal for SCI repair, as it facilitates axons reaching their target directly while minimizing aberrant connections.

In summary, the proposed combinational therapy involving α9-integrin and fibrin gel biomaterials incorporating the AEIDGIEL peptide holds promise for addressing the complex challenges of SCI and promoting effective neural regeneration, laying the foundation for further *in vivo* research. The introduction of fibrin gel with AEIDGIEL peptide to fill the lesion site could prevent cavity expansion, protect neurons from toxic substances at the lesion site, stabilize the spinal cord and reduce its susceptibility to tissue collapse, and serve as a scaffold to facilitate the migration of regenerating axons along the lesion. Fibrin gel has been utilized in both *in vitro* and *in vivo* murine models for SCI repair. Its swelling ratio, rheological properties, mechanical stiffness, as well as fiber structure and pore size, have been thoroughly characterized (Urech et al., 2005) and it demonstrated excellent biocompatibility and non-toxicity of the gel or its degradation products. Another major advantage of the fibrin gel is its ability to be injected as a liquid and solidify *in situ*, conforming to the lesion site’s shape. Our *in vitro* study involved a 2D culture where primary cells were seeded on the gel surface as a proof of concept of the compatibility of this combinatorial strategy and the incorporation of the AEIDGIEL peptide. For *in vivo* use, further optimization of the fibrin gel’s properties is necessary, including achieving the appropriate stiffness (matching the 1.2 kPa of spinal tissue), a suitable porous microstructure to support 3D axonal extension and an appropriate degradation rate. Accurately adjusting the physicochemical properties of the biomaterial is crucial, as these properties influence repair cellular responses such as immunomodulation and innate neurotrophic signaling (Woods et al., 2022). The stiffness and porosity can be easily adjusted by varying the concentrations of fibrinogen and thrombin (Herbert et al., 1998). However, controlling the degradation rate remains a major challenge. In our study, the fibrin gel maintained its integrity for only 5 days *in vitro* before beginning to degrade. For *in vivo* applications, it is essential for the gel to remain functional for at least 6 weeks—the time needed for axons to traverse the lesion site—highlighting a significant gap between our current results and the desired outcome. Future efforts will focus on optimizing the gel’s chemical composition to regulate degradation through innovative strategies, such as incorporating engineered aprotinin (Lorentz et al., 2011) and proceed with *in vivo* studies.

## Data Availability

The datasets presented in this study can be found in online repositories. The names of the repository/repositories and accession number(s) can be found below: https://doi.org/10.5281/zenodo.13142847.

## Footnotes

1
https://arriveguidelines.org/
