# Supplementary material for: Combined strategy of α 9-integrin transduction and AEIDGIEL peptide-functionalized fibrin gel biomaterials to promote mature DRG neurite growth
Source: Front Cell Neurosci. 2025 Apr 1;19:1568004. doi: 10.3389/fncel.2025.1568004 (PMC11996794; doi:10.3389/fncel.2025.1568004)
Supplement: Supplementary file 1 [file Data_Sheet_1.PDF]

Supplementary Material

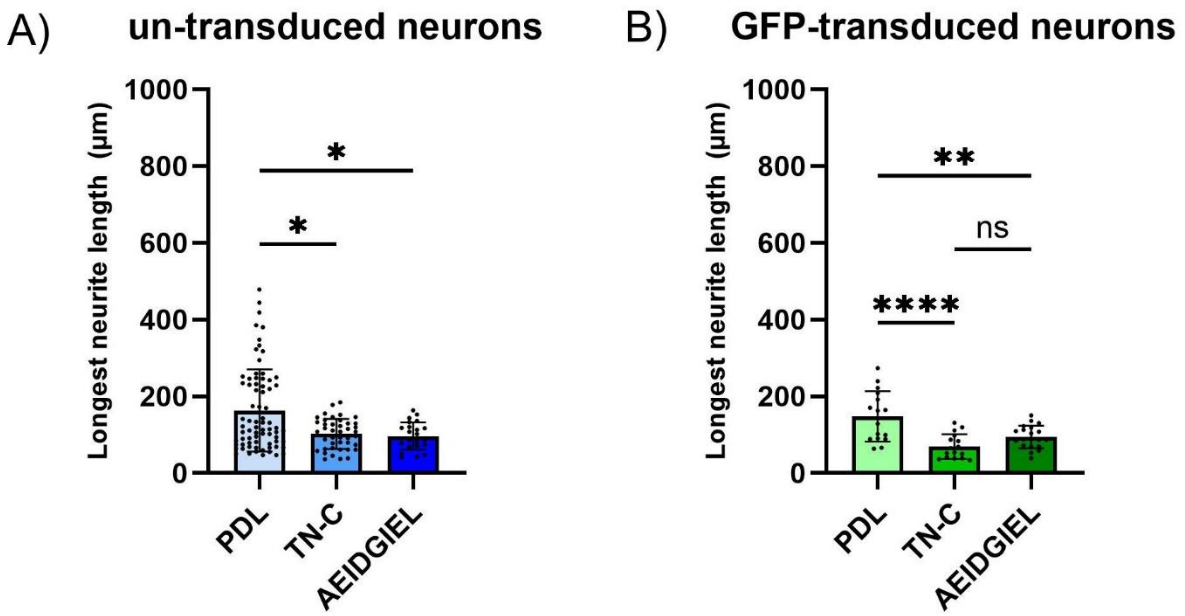

Supplementary Figure 1. WT DRG neurite growth of control neurons growing on PDL, TN-C or AEIDGIEL coated coverslips. Quantification of the longest neurite (µm) growing from A) Un-transduced neurons, B) GFP-transduced neurons. The longest neurite of each neuron was quantified from at least three separate experiments, with data representing individual neurites. Results indicate that both TN-C protein and AEIDGIEL peptide create neurite growth inhibitory environment. * $p < 0.05$, ** $p < 0.01$, **** $p < 0.0001$. Error bars indicate SD.

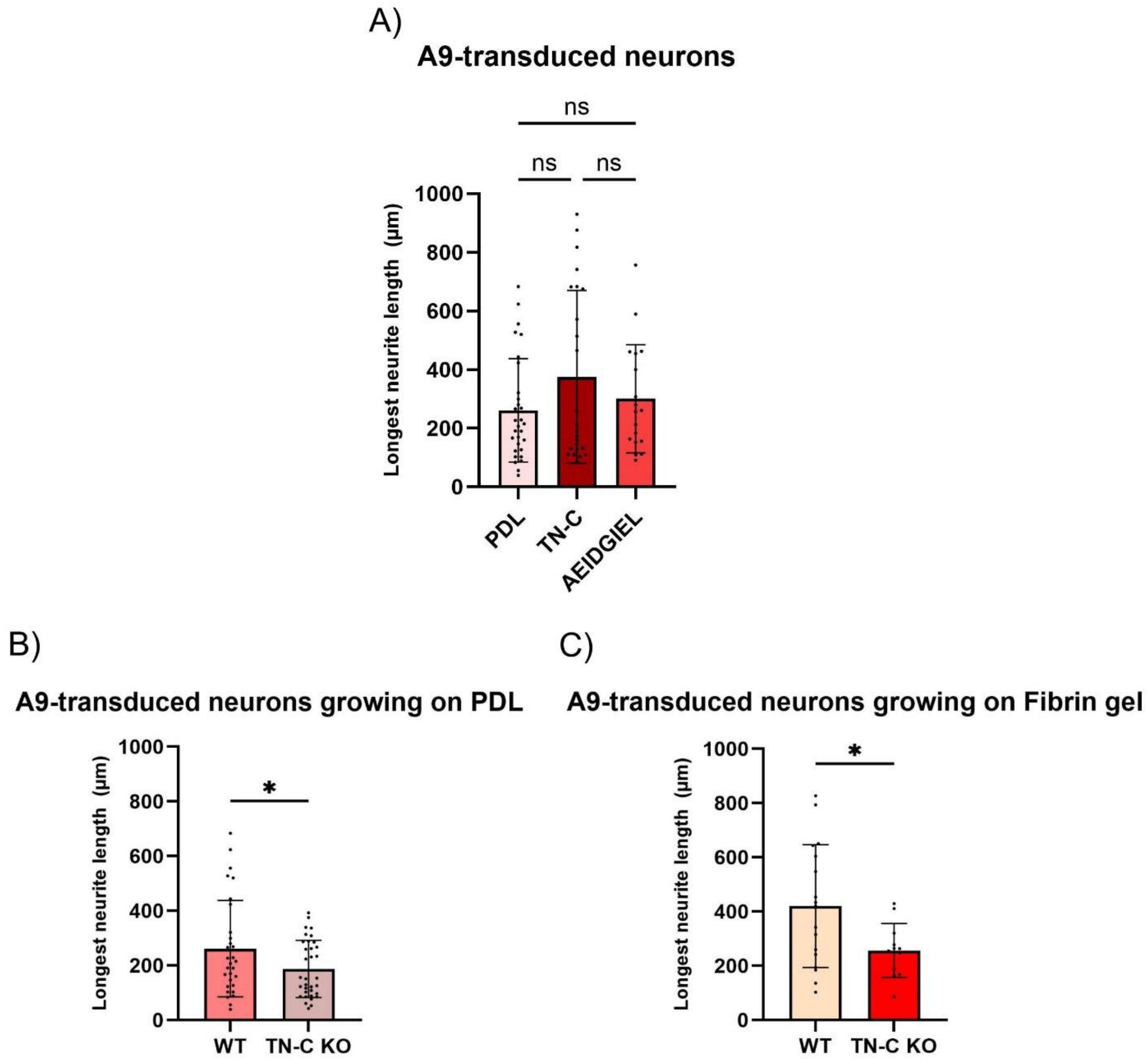

Supplementary Figure 2. WT and TN-C KO DRGs transduced with AAV-α9-V5 growing on different surfaces. The longest neurite of each neuron was quantified from at least three separate experiments, with data representing individual neurites. A) WT DRG neurite growth of a9-transduced neurons growing on PDL, TN-C or AEIDGIEL coated coverslips. Results indicate that a9-transduced neurons grow equally long neurites irrespective of the coverslips coating. B) A9-transduced neurons growing on PDL-coated coverslips from WT and TN-C KO mice. Data indicates that TN-C KO a9-transduced neurons grow shorter neurites than WT neurons on PDL-coated coverslips because the integrin is not activated. C) A9-transduced neurons growing on fibrin gels from WT and TN-C KO mice. Data indicates that TN-C KO a9-transduced neurons grow shorter neurites than WT neurons on fibrin gels because the integrin is not activated. * $p < 0.05$. Error bars indicate SD.

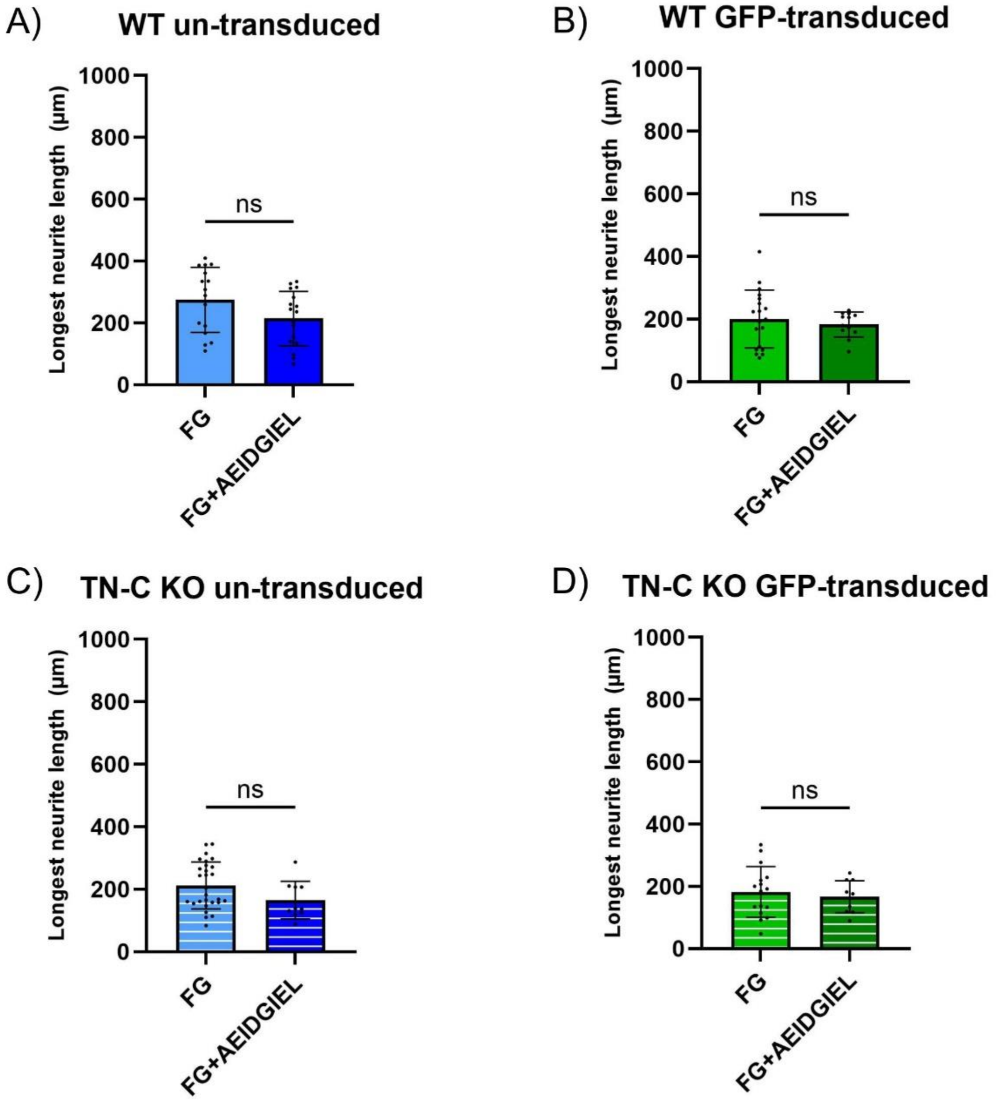

Supplementary Figure 3. Control neurons growing on fibrin gels and AEIDGIEL-modified fibrin gels from WT and TN-C KO mice. The longest neurite of each neuron was quantified from at least three separate experiments, with data representing individual neurites. Data indicates that AEIDGIEL peptide incorporated into the fibrin gel creates just a slightly but not significant inhibitory environment for A) WT un-transduced, B) WT GFP-transduced, C) TN-C KO un-transduced and D) TN-C KO GFP-transduced neurons. Error bars indicate SD.
